# Sp1-Mediated Prdx6 Upregulation Leads to Clasmatodendrosis by Increasing Its aiPLA2 Activity in the CA1 Astrocytes in Chronic Epilepsy Rats

**DOI:** 10.3390/antiox11101883

**Published:** 2022-09-23

**Authors:** Ji-Eun Kim, Duk-Shin Lee, Tae-Cheon Kang

**Affiliations:** Department of Anatomy and Neurobiology, Institute of Epilepsy Research, College of Medicine, Hallym University, Chuncheon 24252, Korea

**Keywords:** astrocyte, autophagy, CDDO-Me, MJ33, MMA, NAC, Nrf2

## Abstract

Clasmatodendrosis is an autophagic astroglial degeneration (a non-apoptotic (type II) programmed cell death) whose underlying mechanisms are fully understood. Peroxiredoxin-6 (Prdx6), the “non-selenium glutathione peroxidase (NSGPx)”, is the only member of the 1-cysteine peroxiredoxin family. Unlike the other Prdx family, Prdx6 has multiple functions as glutathione peroxidase (GPx) and acidic calcium-independent phospholipase (aiPLA2). The present study shows that Prdx6 was upregulated in CA1 astrocytes in chronic epilepsy rats. 2-Cyano-3,12-dioxo-oleana-1,9(11)-dien-28-oic acid methyl ester (CDDO-Me) and N-acetylcysteine (NAC, a precursor of glutathione) ameliorated clasmatodendrosis accompanied by reduced Prdx6 level in CA1 astrocytes. Specificity protein 1 (Sp1) expression was upregulated in CA1 astrocyte, which was inhibited by mithramycin A (MMA). MMA alleviated clasmatodendrosis and Prdx6 upregulation. Sp1 expression was also downregulated by CDDO-Me and NAC. Furthermore, 1-hexadecyl-3-(trifluoroethgl)-sn-glycerol-2 phosphomethanol (MJ33, a selective inhibitor of aiPLA2 activity of Prdx6) attenuated clasmatodendrosis without affecting Prdx6 expression. All chemicals shortened spontaneous seizure duration but not seizure frequency and behavioral seizure severity in chronic epilepsy rats. Therefore, our findings suggest that Sp1 activation may upregulate Prdx6, whose aiPLA2 activity would dominate over GPx activity in CA1 astrocytes and may lead to prolonged seizure activity due to autophagic astroglial degeneration.

## 1. Introduction

Epilepsy is a common chronic neurological disorder manifested by unprovoked recurrent seizures. Temporal lobe epilepsy (TLE) frequently shows poor responses to antiepileptic drugs (AEDs). Aberrant hypersynchronous paroxysmal neuronal discharges in TLE patients lead to further neuronal damage in the various brain regions, especially in the hippocampus (hippocampal sclerosis), which results in secondary symptoms such as cognitive defects and mood disorders [1,2].

Astrocytes maintain brain homeostasis by regulating the clearance of extracellular glutamate and K^+^, brain–blood barrier (BBB) permeability, and neuronal metabolism [3]. In addition, astrocytes take charge of oxidative defense systems in the brain, which is regulated by the nuclear factor erythroid-related factor 2 (Nrf2) [4]. In general, it seems that astrocytes are more invulnerable to harmful stress than neurons. After insults, they are activated and transformed into hypertrophy and hyperplasia (reactive astrogliosis). However, astrocytes are also degenerated in mesial temporal structures of the rat brain following status epilepticus (SE) in spatiotemporal-specific patterns. Briefly, SE-induced astroglial degeneration is observed in the stratum radiatum of the CA1 region of the hippocampus proper, molecular layer of the dentate gyrus, and the piriform cortex, followed by reactive astrogliosis originating from gliogenesis or in situ proliferation [5]. These SE-induced astroglial degenerations demonstrate three distinct patterns based on underlying mechanisms independent of hemodynamics. (1) One is vasogenic edema-induced necrotic astroglial degeneration in piriform cortex [6,7,8]. (2) Another is astroglial apoptosis in the molecular layer of the dentate gyrus [9,10,11]. (3) The other is clasmatodendrosis in the stratum radiatum of the CA1 region of the hippocampus proper [12,13]. Therefore, it is likely that anatomical and physiological properties and vulnerabilities of astrocytes in response to SE may be different from the distinct brain regions [6,7,8,9,10,11,12,13].

Among the types of SE-induced astroglial degeneration, clasmatodendrosis is slowly developed and thus observed in chronic epilepsy rats (>4 weeks after SE). Clasmatodendrosis is first described by Alzheimer as an astroglial injury characterized by extensive swollen and vacuolized cell bodies with disintegrated/beaded processes, and later termed by Cajal [14,15]. Unlike reactive astrocytes, clasmatodendritic astrocytes show vacuolized edematous cell bodies, short blunt processes, glial fibrillary acidic protein (GFAP) tangles, and nuclear dissolution with the preservation of cell outline. At first, we reported that clasmatodendrosis would be coagulative necrosis of astrocytes since vacuolization in the cytoplasm is an early necrotic feature [12]. Later, we found that vacuoles in clasmatodendritic astrocytes are active lysosomes indicating activation of the autophagic process [13,16]. Other investigators have confirmed that clasmatodendritic astrocytes are ubiquitin proteasome system (UPS)-mediated astroglial degeneration under various pathological conditions [17,18]. Thus, clasmatodendrosis is a non-apoptotic (type II) programmed cell death induced by aberrant and dysregulated autophagy [19]. Furthermore, clasmatodendrosis is aggravated by spontaneous seizures and affects seizure duration, but not seizure frequency and behavioral seizure severity in chronic epilepsy rats [5,20]. However, the underlying mechanisms of clasmatodendrosis are largely unknown, although the impaired bioenergetics induced by acidity and/or energy-consuming events due to the prolonged heat shock protein (HSP) 25 expression cause clasmatodendrosis [13,21,22].

On the other hand, peroxiredoxin-6 (Prdx6), the “non-selenium glutathione peroxidase (NSGPx)”, is the only member of the 1-cysteine peroxiredoxin family. Unlike the other Prdx family, Prdx6 has multiple functions as glutathione peroxidase (GPx), acidic calcium-independent phospholipase (aiPLA2), and lysophosphatidylcholine acyl transferase (LPCAT) [23]. Since Prdx6 is dominantly expressed in the astrocytes and not much in the neurons, it participates in oxidative defense mechanisms of astrocytes [24]. Indeed, Prdx6 serves as a marker for oxidative stress in astrocytes, which is negatively regulated by Nrf2 [25,26]. Recently, we have reported that Nrf2 is downregulated in astrocytes and 2-cyano-3,12-dioxo-oleana-1,9(11)-dien-28-oic acid methyl ester (CDDO-Me; RTA 402, an Nrf2 activator) attenuates clasmatodendrosis in chronic epilepsy rats by increasing Nrf2 expression and its nuclear accumulation [20]. Therefore, it is noteworthy exploring whether clasmatodendritic degeneration in the epileptic hippocampus would be closely relevant to the altered Prdx6 functionality in response to oxidative stress, although the profiles of Prdx6 expression in the epileptic hippocampus have not been reported yet.

Here, we demonstrate that Prdx6 was upregulated in CA1 astrocytes showing the decreased Nrf2 level in chronic epilepsy rats. CDDO-Me attenuated clasmatodendrosis and Prdx6 upregulation, accompanied by the increased Nrf2 expression. Unexpectedly, N-acetylcysteine (NAC), a precursor of glutathione (GSH), ameliorated clasmatodendrosis and the increased Prdx6 level in CA1 astrocytes without altering the Nrf2 level. Mithramycin A (MMA)-induced specificity protein 1 (Sp1) inhibition mitigated clasmatodendrosis and Prdx6 upregulation concomitant with unaffected Nrf2 level. Both CDDO-Me and NAC also reduced Sp1 levels in the hippocampus of epileptic rats. Furthermore, 1-hexadecyl-3-(trifluoroethgl)-sn-glycerol-2 phosphomethanol (MJ33, a selective inhibitor of aiPLA2 activity of Prdx6) alleviated clasmatodendrosis without changing the Prdx6 level. Therefore, our findings suggest that Sp1-mediated aberrant Prdx6 upregulation may lead to clasmatodendrosis by increasing its aiPLA2 activity that would dominate over GPx activity, independent of the Nrf2 signaling pathway.

## 2. Materials and Methods

### 2.1. Experimental Animals and Chemicals

Seven-week-old male Sprague-Dawley (SD) rats were procured and maintained under standard environmental conditions (23–25 °C, 12 h light/dark cycle) with water and food provided *ad libitum*. Experimental protocols were allowed by the Institutional Animal Care and Use Committee of Hallym University (Hallym 2021-30, approval date: 17 May 2021). All reagents were obtained from Sigma-Aldrich (St. Louis, MO, USA), except as noted.

### 2.2. SE Induction and Chronic Epilepsy Model

Animals were treated with LiCl (127 mg/kg, i.p.) 1 day before pilocarpine injection. The next day, 20 min before pilocarpine administration, atropine methylbromide (5 mg/kg i.p.) was given. Rats were injected with pilocarpine (30 mg/kg, i.p.). Two hours after SE onset, diazepam (Valium; Hoffmann-la Roche, Neuilly-sur-Seine, France; 10 mg/kg, i.p.) was treated to control seizure activity and repeated as needed. Control animals received saline substituted for pilocarpine. Animals were video-monitored 8 h a day to select chronic epileptic rats [20,22,27,28].

### 2.3. Drug Trials, Electrode Implantation, and Quantification of Seizure Activity

Control and epileptic rats were implanted with a monopolar electrode (Plastics One, Roanoke, VA, USA) in the right hippocampus (coordinates: 3.8 mm posterior; 2.0 mm lateral; 2.6 mm depth) and a brain infusion kit 1 (Alzet, Cupertino, CA, USA) into the right lateral ventricle (coordinates: 1 mm posterior; 1.5 mm lateral; 3.5 mm depth) under Isoflurane anesthesia (3% induction, 1.5–2% for surgery, and 1.5% maintenance in a 65:35 mixture of N_2_O:O_2_). Some animals were inserted with only a brain infusion kit 1. Thereafter, an Alzet 1007D osmotic pump (Alzet, Cupertino, CA, USA) containing (1) vehicle (2) CDDO-Me (an Nrf2 activator, 10 μM), (3) MMA (an Sp1 DNA-binding transcriptional inhibitor, 25 μM) and (4) MJ33 (50 μM) is connected. In some vehicle-infused animals, NAC (70 mg/kg) was administered once a day by intraperitoneal (i.p.) over 7 days. The dose of each compound did not show any off-target effects (paralysis, vocalization, food intake, or neuroanatomical damage) [13,27,29]. The correct location of the infusion site was confirmed during brain sections and sampling tissues for Western blot. The electrode and infusion needle were fixed to the skull with dental cement. Three days after surgery, an electroencephalogram (EEG) was recorded 2 h a day at the same time over 4 days. Behavioral seizure severity was also measured according to Racine’s scale [30]. After recording, animal tissues were used for Western blot [20].

### 2.4. Western Blot

Animals were anesthetized with urethane anesthesia (1.5 g/kg, i.p.) and decapitated. The brains were quickly removed and coronally cut 1 mm thickness (approximately 3–4 mm posterior to the bregma) using rodent brain matrix (World Precision Instruments, Sarasota, FL, USA) on ice. In turn, the stratum radiatum of the CA1 region of the dorsal hippocampus was rapidly collected in cold (4 °C) artificial cerebrospinal fluid under a stereomicroscope [11,20]. The selected tissues were homogenized, and protein concentration was calibrated with a Micro BCA Protein Assay Kit (Pierce Chemical, Rockford, IL, USA). Following electrophoresis, proteins were transferred to nitrocellulose membranes. Membranes were blocked with 2% bovine serum albumin (BSA) in Tris-buffered saline (TBS; in mM 10 Tris, 150 NaCl, pH 7.5, and 0.05% Tween 20) for 1 h at room temperature and reacted with primary antibodies (Supplementary Table S1) overnight at 4 °C and secondary antibodies for 1 h at room temperature. After the chemiluminescence reaction (ECL Western Blotting System, GE Healthcare Korea, Seoul, South Korea), immunobands were detected and quantified on an ImageQuant LAS4000 system (GE Healthcare Korea, Seoul, South Korea).

### 2.5. Immunohistochemistry

Animals were anesthetized with urethane anesthesia (1.5 g/kg, i.p.) and perfused with normal saline followed by 4% paraformaldehyde in 0.1 M phosphate buffer (PB, pH 7.4). After post-fixation with the same fixative and cryoprotection with 30% sucrose overnight, 30 μm thick coronal sections were made using a cryostat. Sections were blocked with 3% bovine serum albumin in PBS for 30 min at room temperature and incubated with a cocktail solution containing primary antibodies (Supplementary Table S1) in PBS containing 0.3% Triton X-100 overnight at room temperature. Thereafter, tissue sections were reacted with Cy2- or Cy3-conjugated secondary antibodies. A negative control test was performed with pre-immune serum instead of the primary antibody. The random-selected 5 areas/animals (300 μm^2^/area) in the hippocampus (5 sections from each animal, *n* = 7 in each group) were selected and measured fluorescent intensity by using AxioVision Rel. 4.8 and ImageJ software. Fluorescent intensity was normalized by setting the mean background. For quantification of clasmatodendritc astrocytes, sections (10 sections per each animal) were captured, and vacuolized astrocytes were counted in areas of interest (1 × 10^4^ μm^2^) selected from the stratum radiatum of the CA1 region using AxioVision Rel. 4.8 Software (Carl Zeiss Korea, Seoul, South Korea) [20,27].

### 2.6. Data Analysis

After Shapiro–Wilk *W*-test for evaluating normality, data were analyzed using Student’s *t*-test (comparison between two groups), one-way analysis of variance (ANOVA), followed by Bonferroni’s *post hoc* comparisons (comparison among three or more independent groups). Kruskal–Wallis test (non-parametric comparison of seizure frequency, duration, and severity among five groups) was also applied. A *p*-value less than 0.05 was considered statistically significant.

## 3. Results

### 3.1. Prdx6 Is Upregulated in CA1 Astrocytes in the Epileptic Hippocampus

**Figure 1:**
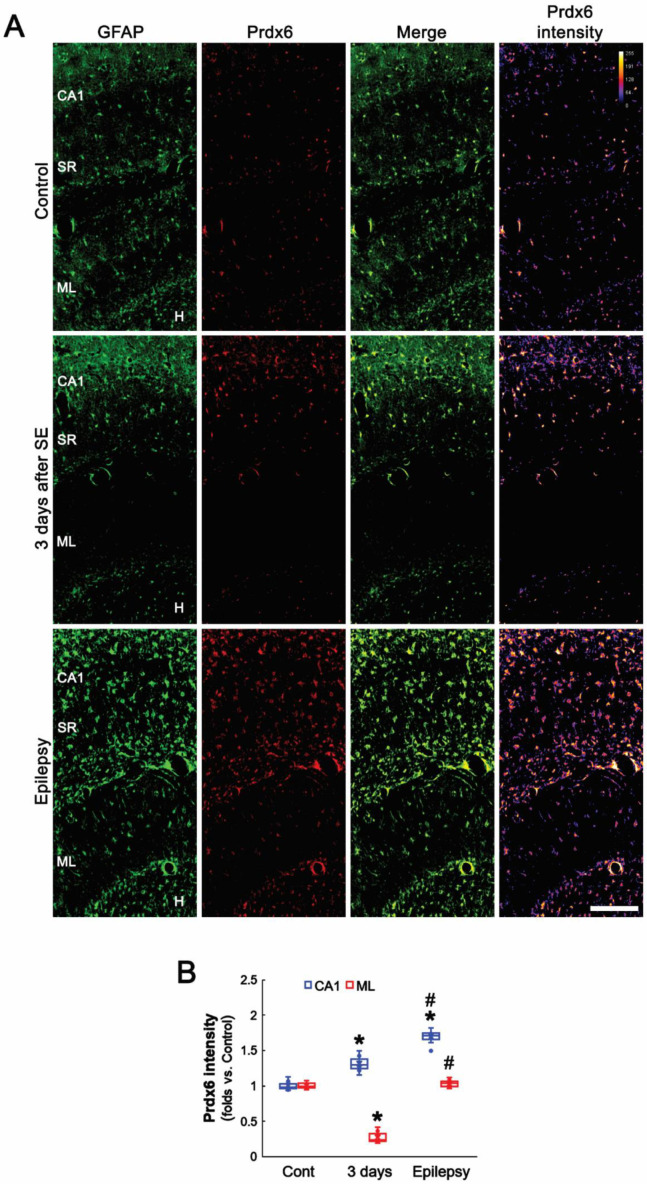
**Effects of SE onPrdx6expression in the rat hippocampus.** Prdx6 expression is mainly observed in astrocytes (GFAP, an astroglial marker) under physiological conditions but not in CA1 pyramidal cells (CA1). Three days after SE, Prdx6 expression increases in the stratum radiatum (SR) of the CA1 region but decreases in the molecular layer (ML) of the dentate gyrus due to astroglial loss. In chronic epilepsy rats, Prdx6 expression upregulates in the stratum radiatum of the CA1 region and the hilus (H), and restores to the control level in the molecular layer of the dentate gyrus. (**A**) Representative photos of Prdx6 expression and its intensity. Bar = 250 μm. (**B**) Quantification of Prdx6 intensity in the stratum radiatum of the CA1 region and the molecular layer of the dentate gyrus (*^,#^ *p* < 0.05 vs. control animals and 3-days post-SE animals, respectively; one-way ANOVA with Bonferroni’s *post hoc* test, *n* = 7, respectively).

Consistent with previous studies [24,31,32,33], the present study showed that Prdx6 expression was restricted to astrocytes in the hippocampus of control animals (Figure 1A). Three days after SE, Prdx6 was upregulated to 1.31-fold of the control level in astrocytes within the CA1 region. In chronic epilepsy rats, Prdx6 expression was elevated in CA1 astrocytes more than 3 days post-SE animals (*F*_(2,18)_ = 83.36, *p* < 0.001, *n* = 7, respectively, one-way ANOVA with Bonferroni’s *post hoc* test; Figure 1A,B). In the molecular layer of the dentate gyrus, the Prdx6 level was reduced to 0.27-fold of the control level 3 days after SE due to the massive astroglial loss and restored to the control level in newly generated astrocytes [9] (*F*_(2,18)_ = 318.33, *p* < 0.001, *n* = 7, respectively, one-way ANOVA with Bonferroni’s *post hoc* test; Figure 1A,B).

### 3.2. CDDO-Me and NAC Attenuate Clasmatodendrosis Accompanied by Prdx6 Downregulation

**Figure 2:**
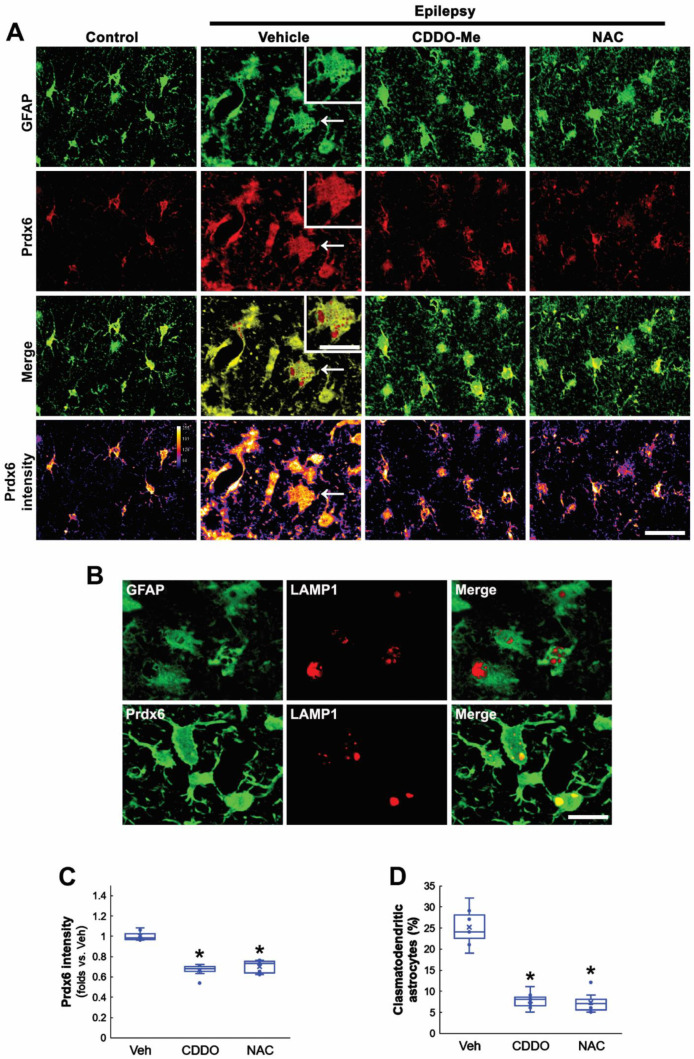
**Effects of CDDO-Me and NAC on Prdx6 expression and clasmatodendritic degeneration of CA1 astrocytes in chronic epilepsy rats.** As compared to control animals, Prdx6 expression is upregulated in reactive CA1 astrocytes and clasmatodendritic CA1 astrocytes (arrows). As compared to vehicle (Veh), CDDO-Me and NAC diminish Prdx6 expression and attenuate clasmatodendrosis of CA1 astrocytes. (**A**) Representative photos of Prdx6 expression and its intensity. Insertions are high magnification of arrows. Bar = 25 and 12.5 μm (insertions). (**B**) Representative photos of clasmatodendritic CA1 astrocytes containing lysosome-associated membrane protein 1 (LAMP1)-positive vacuoles. Prdx6 expression is also detected in LAMP1-positive vacuoles. (**C**,**D**) Quantifications of Prdx6 intensity (C) and clasmatodendritic degeneration (D) in CA1 astrocytes (* p < 0.05 vs. vehicle; one-way ANOVA with Bonferroni’s *post hoc* test, *n* = 7, respectively).

Since oxidative stress causes clasmatodendrosis [13,20,27], we applied two different antioxidants, CDDO-Me and NAC, in chronic epilepsy rats and investigated their effects on Prdx6 expression and autophagic astroglial degeneration. In control animals, Prdx6 expression was mainly observed in the cytoplasm of CA1 astrocytes (Figure 2A). In the epileptic hippocampus, Prdx6 expression was upregulated in most CA1 astrocytes (Figure 2A). Prdx6 intensity in clasmatodendritic astrocytes was similar to that in reactive astrocytes (Figure 2A). Prdx6 expression in clasmatodendritic astrocytes was also detected in vacuoles showing lysosome-associated membrane protein 1 (LAMP1) signals, indicating lysosomal Prdx6 localization (Figure 2A,B). Both CDDO-Me and NAC significantly reduced Prdx6 level in CA1 astrocyte (*F*_(2,18)_ = 66.64, *p* < 0.001, *n* = 7, respectively, one-way ANOVA with Bonferroni’s *post hoc* test; Figure 2A,C) and effectively attenuated vacuolized degeneration of CA1 astrocytes in the epileptic hippocampus (*F*_(2,18)_ = 71.19, *p* < 0.001, *n* = 7, respectively, one-way ANOVA with Bonferroni’s *post hoc* test; Figure 2A,D). These findings indicate that Prdx6 upregulation may be an adaptive response against oxidative stress in the cytoplasm and be relevant to astroglial autophagic degeneration.

### 3.3. NAC Inhibits Prdx6 Upregulation Independent of Nrf2

Nrf2 activation induces Prdx6 upregulation [34], and Prdx6 is required for Nrf2-mediated protection against oxidative stress in astrocytes [35]. In a previous study, we reported that Nrf2 expression is reduced in clasmatodendritic CA1 astrocytes in epileptic rats. CDDO-Me attenuates this autophagic astroglial degeneration accompanied by Nrf2 upregulation [20]. Therefore, we validated the effects of CDDO-Me and NAC on Nrf2 expression in clasmatodendritic astrocytes.

**Figure 3:**
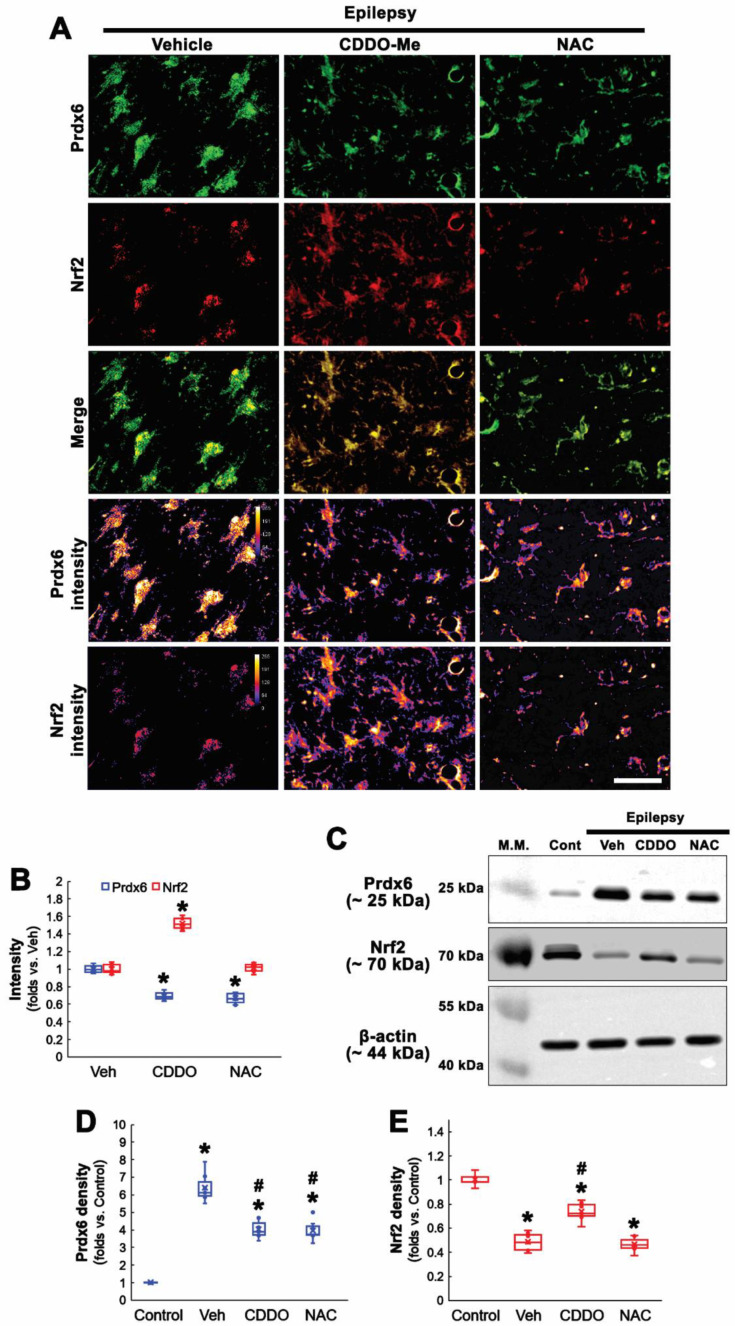
**Effects of CDDO-Me and NAC on Prdx6 and Nrf2 expressions in CA1 astrocytes in chronic epilepsy rats.** As compared to control animals, Nrf2 expression is decreased in CA1 astrocytes of chronic epilepsy rats. As compared to vehicle (Veh), CDDO-Me decreases Prdx6 expression concomitant with Nrf2 upregulation, while NAC diminishes Prdx6 expression without altering Nrf2 expression. (**A**) Representative photos of Prdx6 and Nrf2 expressions and their intensities. Bar = 25 μm. (**B**) Quantifications of Prdx6 and Nrf2 intensities in CA1 astrocytes (* p < 0.05 vs. vehicle; one-way ANOVA with Bonferroni’s *post hoc* test, *n* = 7, respectively). (**C**) Representative Western blot of Prdx6 and Nrf2 in the CA1 region. (**D**,**E**) Quantifications of Prdx6 (D) and Nrf2 (E) densities in the CA1 region (*^,#^ *p* < 0.05 vs. control animals and vehicle-treated animals, respectively; one-way ANOVA with Bonferroni’s *post hoc* test, *n* = 7, respectively).

Consistent with our previous study [20], CDDO-Me increased Nrf2 level to 1.52-fold of vehicle level in CA1 astrocytes, while NAC did not (*F*_(2,18)_ = 170.78, *p* < 0.001, *n* = 7, respectively, one-way ANOVA with Bonferroni’s *post hoc* test; Figure 3A,B). Compatible with immunohistochemistry, Western blot data revealed that Prdx6 expression was upregulated in the epileptic hippocampus, which was attenuated by CDDO-Me and NAC (*F*_(3,24)_ = 111.17, *p* < 0.001, *n* = 7, respectively, one-way ANOVA with Bonferroni’s *post hoc* test; Figure 3C,D and Supplementary Figure S1). In contrast, Nrf2 was decreased in the epileptic hippocampus, which was enhanced by CDDO-Me, but not NAC (*F*_(3,24)_ = 102.87, *p* < 0.001, *n* = 7, respectively, one-way ANOVA with Bonferroni’s *post hoc* test; Figure 3C,E and Supplementary Figure S1). These findings indicate that NAC may ameliorate oxidative stress-induced Prdx6 upregulation in CA1 astrocytes independent of the Nrf2-mediated signaling pathway.

### 3.4. Sp1 Inhibition Ameliorates Prdx6 Upregulation and Clasmatodendrosis in CA1 Astrocytes

**Figure 4:**
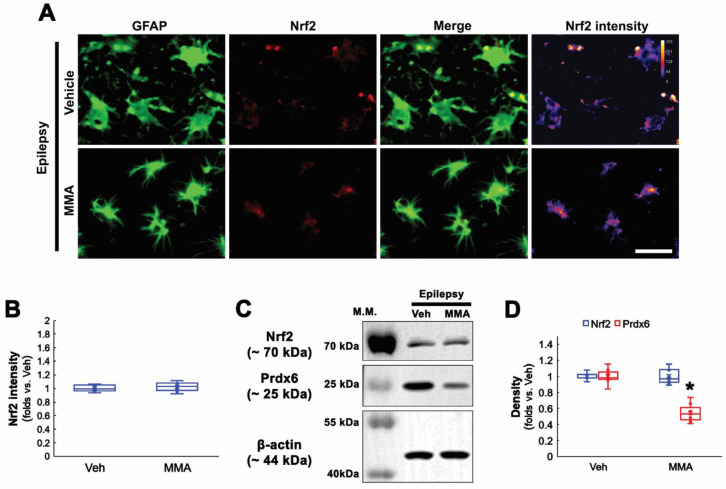
**Effects of MMA on Prdx6 and Nrf2 expressions in CA1 astrocytes in chronic epilepsy rats.** As compared to vehicle (Veh), MMA decreases Prdx6 expression in the epileptic hippocampus without affecting Nrf2 expression. (**A**) Representative photos of Nrf2 expression and its intensity. Bar = 25 μm. (**B**) Quantifications of Nrf2 intensity in CA1 astrocytes (*n* = 7, respectively). (**C**) Representative Western blot of Nrf2 and Prdx6 in the CA1 region. (**D**) Quantifications of Nrf2 and Prdx6 densities in the CA1 region (* p < 0.05 vs. vehicle, respectively; Student *t*-test, *n* = 7, respectively).

**Figure 5:**
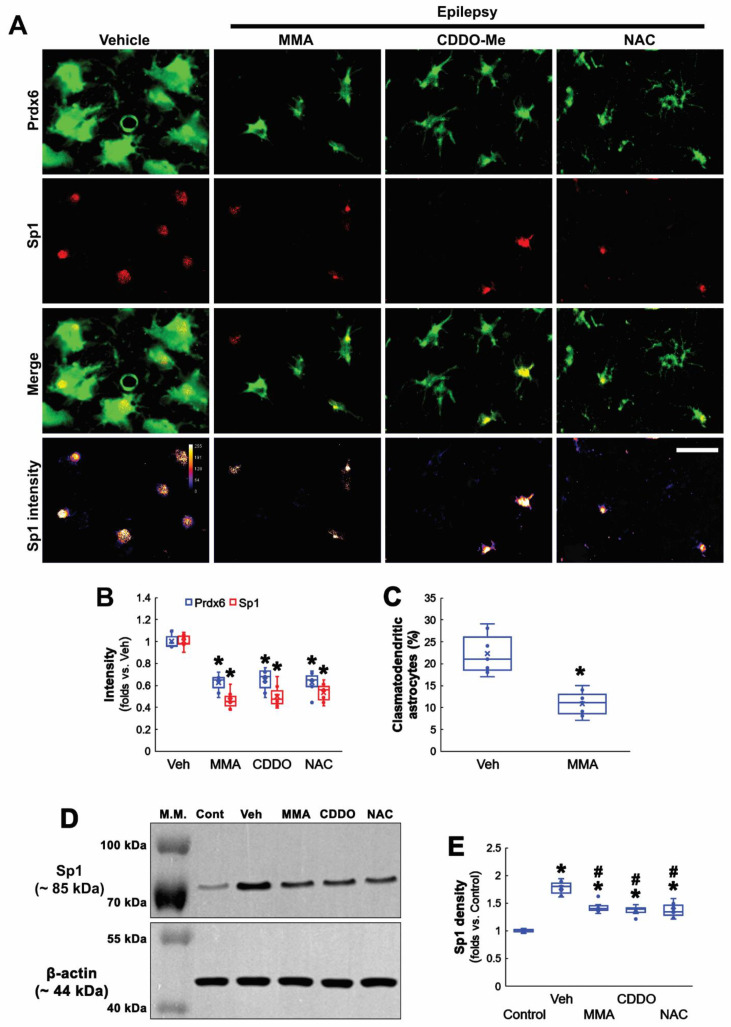
**Effects of MMA, CDDO-Me, and NAC on Prdx6 and Sp1 expressions and clasmatodendritic degeneration of CA1 astrocytes in chronic epilepsy rats.** As compared to vehicle (Veh), MMA, CDDO-Me, and NAC decrease Prdx6 expression and attenuate clasmatodendritic degeneration accompanied by Sp1 downregulation. (**A**) Representative photos of Prdx6 and Sp1 expressions and their intensities. Bar = 25 μm. (**B**) Quantifications of Prdx6 and Sp1 intensities in CA1 astrocytes (* p < 0.05 vs. vehicle; one-way ANOVA with Bonferroni’s *post hoc* test, *n* = 7, respectively). (**C**) Quantification of clasmatodendritic degeneration in CA1 astrocytes (* p < 0.05 vs. vehicle; Student *t*-test, *n* = 7, respectively). (**D**) Representative Western blot of Sp1 expression in the CA1 region. (**E**) Quantification of Sp1 density in the CA1 region (*^,#^ p < 0.05 vs. control animals and vehicle-treated animals, respectively; one-way ANOVA with Bonferroni’s *post hoc* test, *n* = 7, respectively).

Seizures increase Sp1 activity, which is one of the upstream regulators for clasmatodendrosis [13,36]. Furthermore, Sp1 overexpression reduces Nrf2 protein level [37], and CDDO-Me decreases Sp1 expression [38]. Therefore, it is likely that Sp1 activation would lead to Prdx6 upregulation in CA1 astrocytes within the epileptic hippocampus. To confirm this, we applied MMA (an Sp1 DNA-binding transcriptional inhibitor) [13,39] in chronic epilepsy rats. As compared to vehicles, MMA did not affect Nrf2 levels in CA1 astrocytes within the epileptic hippocampus (Figure 4A–D and Supplementary Figure S1). However, MMA effectively reduced Prdx6 level in the epileptic hippocampus (*t*_(12)_ = 7.71, *p* < 0.001, *n* = 7, respectively, Student *t*-test; Figure 4C,D and Supplementary Figure S1). Furthermore, MMA diminished the Sp1 expression levels in CA1 astrocytes. CDDO-Me and NAC also decreased Sp1 expression in CA1 astrocytes, accompanied by the reduced Prdx6 level (*F*_(3,24)_ = 60.8, *p* < 0.001, *n* = 7, respectively, one-way ANOVA with Bonferroni’s *post hoc* test; Figure 5A,B). Similar to the cases of CDDO-Me and NAC, MMA attenuated autophagic degeneration of CA1 astrocytes (*t*_(12)_ = 5.31, *p* < 0.001, *n* = 7, respectively, Student *t*-test; Figure 5A,C and Supplementary Figure S1). Western blot data revealed that Sp1 expression was upregulated to 1.78-fold of control level in the epileptic hippocampus, which was ameliorated by MMA, CDDO, and NAC (*F*_(4,30)_ = 47.22, *p* < 0.001, *n* = 7, respectively, one-way ANOVA with Bonferroni’s *post hoc* *test*; Figure 5D,E). These findings indicate that Sp1 upregulation may increase Prdx6 expression, which would lead to clasmatodendrosis, independent of Nrf2.

### 3.5. MJ33 Ameliorates Clasmatodendritic Degeneration in the Epileptic Hippocampus

**Figure 6:**
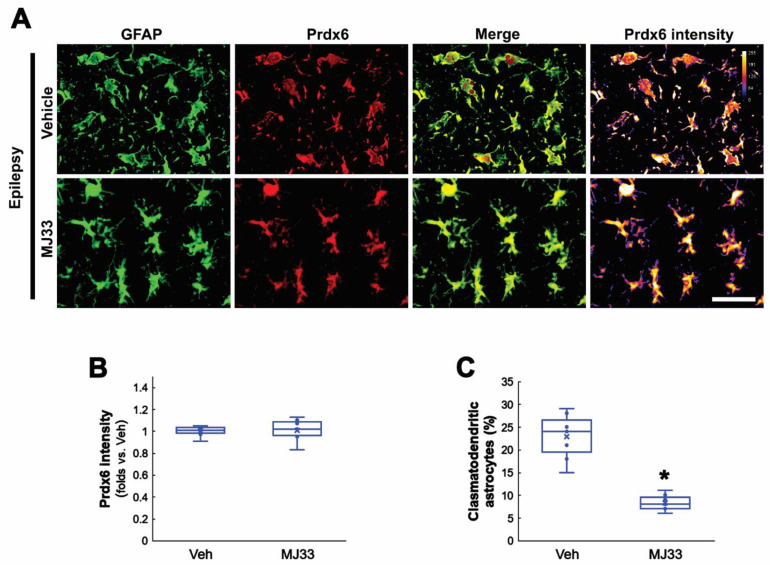
**Effects of MJ33 on Prdx6 expression in CA1 astrocytes in chronic epilepsy rats.** As compared to vehicle (Veh), MJ33 does not affect Prdx6 expression but attenuates clasmatodendritic degeneration in CA1 astrocytes. (**A**) Representative photos of Prdx6 expression and its intensity. Bar = 25 μm. (**B**) Quantifications of Prdx6 intensity in CA1 astrocytes (*n* = 7, respectively). (**C**) Quantification of clasmatodendritic degeneration in CA1 astrocytes (* p < 0.05 vs. vehicle; Student *t*-test, *n* = 7, respectively).

In the present study, Sp1 inhibition attenuated clasmatodendrosis in the epileptic hippocampus by reducing the Prdx6 expression level. Considering the GPx properties of Prdx6, therefore, the remaining question is how upregulated Prdx6 would lead to autophagic astroglial degeneration rather than protect them from clasmatodendrosis. Aforementioned, Prdx6 is a bifunctional enzyme that has aiPLA2 activity as well as GPx function [16]. Since the aiPLA2 activity of Prdx6 is harmful to cell viability [40,41], it is likely that increased aiPLA2 activity of upregulated Prdx6 would be involved in clasmatodendrosis. To confirm this hypothesis, we applied MJ33 (a selective inhibitor of aiPLA2 activity of Prdx6) in chronic epilepsy rats. Consistent with a previous study [42], MJ33 did not affect the Prdx6 level in CA1 astrocytes (Figure 6A,B). However, MJ33 alleviated clasmatodendritic degeneration in CA1 astrocytes (*t*_(12)_ = 7.07, *p* < 0.001, *n* = 7, respectively, Student *t*-test; Figure 6A,C). Thus, these findings indicate that upregulated Prdx6 in CA1 astrocytes within the epileptic hippocampus may function as aiPLA2 rather than GPx, which would exacerbate autophagic astroglial degeneration.

### 3.6. Attenuations of Clasmatodendrosis Reduce Seizure Duration, but Not Its Frequency and Severity in Chronic Epilepsy Rats

**Figure 7:**
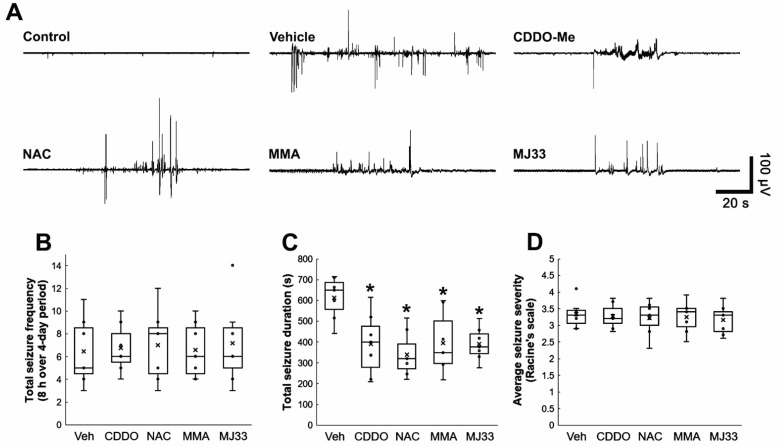
**Effects of CDDO-Me, NAC, MMA, and MJ33 on spontaneous seizure activity in epileptic rats.** CDDO-Me, NAC, MMA, and MJ33 reduce seizure duration but not seizure frequency and its severity in epileptic rats. (**A**) Representative EEG traces obtained from control and epileptic rats. (**B**–**D**) Quantitative values of total seizure frequency (**B**), total seizure duration (**C**), and seizure severity (**D**) over a 4-day period. Open circles indicate each individual value. Horizontal bars indicate the mean value. Error bars indicate SD (* p < 0.05 vs. vehicle (Veh)-treated animals; Kruskal–Wallis test; *n* = 7, respectively).

Recently, we have reported that the prevention of clasmatodendrosis induced by CDDO-Me decreases seizure duration without affecting seizure frequency and its severity [20]. Thus, we evaluated whether the protective effects of NAC, MMA, and MJ33 against clasmatodendrosis also influence spontaneous seizure activity in epileptic rats over a 4-day period. In vehicle-treated animals, the total seizure frequency was 6.4 ± 2.9/recording session, and the total seizure duration was 611.7 ± 102.8 s. The seizure severity (behavioral seizure core) was 3.3 ± 0.41 (Figure 7A–D). As compared to vehicle, all chemicals including CDDO-Me reduced total seizure duration (*χ^2^*_(4)_ = 12.69, *p* = 0.013, Kruskal–Wallis test, *n* = 7, respectively; Figure 7A,C) without altering total seizure frequency (*χ^2^*_(4)_ = 0.195, *p* = 0.996, Kruskal–Wallis test, *n* = 7, respectively; Figure 7A,B) and seizure severity (*χ^2^*_(4)_ = 0.4, *p* = 0.982, Kruskal–Wallis test, *n* = 7, respectively; Figure 7A,D). These findings indicate that clasmatodendritic degeneration may influence seizure duration, but not its frequency and severity, in chronic epileptic rats.

## 4. Discussion

**Figure 8:**
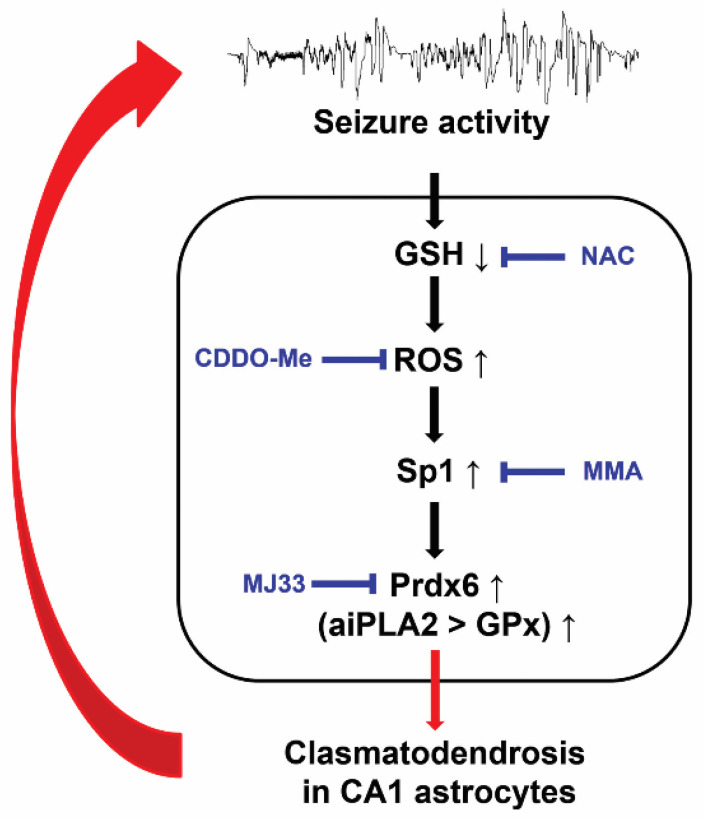
**Scheme of the underlying mechanism of Prdx6-mediated clasmatodendrosis.** GSH deficiency exacerbates oxidative stress, which activates Sp1-mediated Prdx6 upregulation. Subsequently, upregulated Prdx6 acts as aiPLA2 rather than GPx, leading to clasmatodendrosis.

The present study shows that in the epileptic hippocampus, Prdx6 expression was upregulated in CA1 astrocytes. Antioxidants (CDDO-Me and NAC) attenuated clasmatodendrosis accompanied by reduced Prdx6 expression. Sp1 inhibition by MMA downregulated Prdx6 expression in CA1 astrocytes. Furthermore, inhibition of the aiPLA2 activity of Prdx6 by MJ33 ameliorated clasmatodendrosis. Thus, our findings indicate that Prdx6 upregulation was induced by Sp1 activation in response to oxidative stress and that the upregulated Prdx6 may act as aiPLA2 rather than GPx, leading to clasmatodendrosis (Figure 8).

Prdx6 upregulation has been reported as a defensive compensatory reaction to the oxidative damage in patients and animal models of Parkinson’s disease, Alzheimer’s disease, Pick’s disease, amyotrophic lateral sclerosis, glioma, and traumatic brain injury [23,27,29,43,44,45]. In the present study, the Prdx6 level was reduced in the molecular layer of the dentate gyrus 3 days after SE and restored to the control level in chronic epilepsy rats. Recently, it has been reported that Prdx6 inhibits a global increase in [Ca^2+^]_i_ in astrocytes and neurons, which suppresses cell necrosis, especially in the astrocyte population, during oxygen-glucose deprivation/reoxygenation (OGD/R) condition. Prdx6 also attenuates apoptosis induced by OGD/R. These effects of Prdx6 are closely related to the activation of the various kinases abrogating signaling pathways concerning apoptosis, pro-inflammatory cytokine productions, and glutamate receptor expressions [46]. Considering this report [46] and SE-induced astroglial apoptosis followed by gliogenesis in this region [9,10,11], it is likely that Prdx6 downregulation may be related to astroglial apoptosis in the molecular layer of the dentate gyrus and that the newly generated astrocytes in this region may restore Prdx6 expression to control level.

Paradoxically, Prdx6 can also increase the generation of reactive oxygen species (ROS) by activating NADPH oxidase [42,47]. Indeed, Prdx6 overexpression accelerates the development of Parkinson’s disease, Alzheimer’s disease, and experimental autoimmune encephalomyelitis in animal models [45,48,49]. Prdx6 is located in the cytoplasm but after translocated into acidic organelles, such as lysosomes and lysosome-related secretory organelles (lamellar bodies) [50]. aiPLA2 activity of Prdx6 is maximal at pH 4, whereas the GPx activity is expressed at pH 7 [51]. Therefore, Prdx6 acts as aiPLA2 in lysosomes [52]. Compatible with these reports, the present data show that Prdx6 expression was observed in lysosomes as well as the cytoplasm in clasmatodendritic astrocytes. Oxidative stress impairs the enzymatic steps of GSH synthesis induced by intracellular acidosis [53,54], which subsequently leads to clasmatodendritic degeneration via downregulation of GPx1 that catalyzes the reduction in H_2_O_2_ by using GSH [27]. In the brain, astrocytes show dynamic pH changes more than neurons or interstitial space [55]. Intracellular pH in astrocytes falls to 5.2–5.3 in astrocytes under pathophysiological conditions, while it is 7.2–7.3 under physiological conditions. In contrast, the pH of neurons and interstitial space are equilibrated to 6.2 due to impaired ion transporter systems. This intracellular acidosis leads to clasmatodendritic degeneration in astrocytes [21,56]. Considering the low pH requirement for aiPLA2 activity of Prdx6, it is likely that intracellular acidosis in astrocytes may increase aiPLA2 activity of Prdx6 rather than GPx activity in the cytoplasm and subsequently reinforce ROS generation by NADPH oxidase activation [42,47]. Furthermore, ROS-induced hyperoxidation of Prdx6 at cysteine 47 increases aiPLA2 activity but inhibits its GPx activity, which is irreversible [57]. The present study reveals that inhibition of aiPLA2 activity of Prdx6 by MJ33 ameliorated autophagic degeneration of CA1 astrocytes in the epileptic hippocampus, indicating that aiPLA2 activity of Prdx6 may dominate over its GPx activity in clasmatodendritic CA1 astrocytes. Therefore, it is likely that intracellular acidosis and ROS generation may enhance the aiPLA2 activity of Prdx6 in a positive feedback manner, which would exacerbate clasmatodendrosis. Similar to the case of HSP25 [13,20,22], furthermore, our findings suggest the double-edge profiles of Prdx6 in SE-induced astroglial death, which may be a modulator for clasmatodendrosis in the CA1 region as well as astroglial apoptosis in the molecular layer of the dentate gyrus.

Nrf2 is one of the main regulators of cellular redox status whose activity increases under oxidative stress conditions, which is responsible for Prdx6 transcription [58]. Indeed, Nrf2 induction by tert-butylhydroquinone (tBHQ) increases the Prdx6 level in astrocytes [35]. However, Nrf2 activity is also modulated by the aiPLA2 activity of Prdx6 [59]. Nrf2 negatively regulates Prdx6 expression, and inhibition of aiPLA1 activity of Prdx6 by MJ33 abrogates upregulation of Nrf2 in astrocytes following hypoxia-reperfusion injury [26]. Therefore, the role of Nrf2 in Prdx6 transactivation under pathophysiological conditions has still been controversial. Consistent with our previous study [20], the present data show that the Nrf2 level in CA1 astrocytes of chronic epilepsy rats was lower than that of control (normal) animals, which was increased by CDDO-Me. In addition, CDDO-Me diminished Prdx6 and Sp1 expressions in CA1 astrocytes of chronic epilepsy rats. As Nrf2 inhibits Sp1 activation [60], it is likely that CDDO-Me-induced Nrf2 induction would alleviate the Prdx6 expression level by inhibiting Sp1 activity. However, NAC and MMA reduced Prdx6 and Sp1 levels in CA1 astrocytes without inducing Nrf2 upregulation. Sp1 is a transcription factor to regulate expressions of Nrf2 and HSP25, which are involved in clasmatodendrosis [13,20,22,37]. Furthermore, Sp1 inhibition abolishes curcumin-induced Prdx6 upregulation following cerebral ischemia [61]. Since NAC and MMA ameliorated clasmatodendrosis by abrogating Prdx6 upregulation through Sp1 repression, our findings suggest that Sp1 may be one of the upstream regulators of Prdx6 expression in autophagic astroglial degeneration and that Nrf2 may not be essential for Prdx6 suppression in clasmatodendritic astrocytes.

On the other hand, Sp1 activation is required for astroglial activation and proliferation during reactive astrogliosis [62]. Thus, it cannot be excluded the possibility that CDDO-Me, NAC, and MMA would reduce astroglial activation followed by Sp1 and Prdx6 downregulations. Considering the antioxidant properties of CDDO-Me and NAC [20,27], however, it is more plausible that Sp1 repression may be a consequence of the reduced ROS level induced by CDDO-Me and NAC, although MMA may directly abrogate Sp1-mediated astroglial activation. Indeed, CDDO-Me downregulates Sp1 expression [38]. Since Sp1 increases HSP25 expression and promotes upregulation of the negative regulator of ROS (NRROS) that dampens ROS generation in astrocytes [13,63], our findings suggest that Sp1 upregulation in astrocytes may be an adaptive response against oxidative stress through transactivation of various endogenous antioxidant enzymes including Prdx6, while the aberrant increased aiPLA2 activity of Prdx6 may elicit clasmatodendrosis.

In the present study, attenuation of clasmatodendrosis diminished seizure duration without affecting seizure frequency and its severity in chronic epilepsy rats. Regarding that astroglial dysfunctions result in reverberant epileptiform discharges due to impaired K^+^ buffering [64], our findings indicate that clasmatodendrosis in the epileptic hippocampus may lengthen seizure duration rather than ictogenesis. Indeed, aquaporin-4 (AQP4; a water channel) expression is significantly downregulated in clasmatodendritic astrocytes, and AQP4-deleted astrocytes extend seizure duration by impaired K^+^ clearance [12,65,66]. Furthermore, we have reported that the attenuation of clasmatodendrosis by CDDO-Me shortens seizure duration without affecting seizure frequency and its severity [20]. Although the role of clasmatodendrosis in spontaneous seizures is not directly uncovered, it is presumable that K^+^ buffering capacity in the ictal stage may be debased in clasmatodendritic astrocytes, which would retain the duration of synchronous discharges in the epileptic hippocampus.

## 5. Conclusions

The present study reveals for the first time that Sp1 may lead to sustained Prdx6 upregulation in CA1 astrocytes with the epileptic hippocampus. Furthermore, the increased Prdx6 in CA1 astrocytes may act as an aiPLA2 rather than GPx, which would exert autophagic astroglial degeneration. Therefore, our findings suggest that inhibition of the aiPLA2 activity of Prdx6 may be a strategy to protect astrocytes from oxidative stress and ameliorate the duration of spontaneous seizure activity.

## Data Availability

Not applicable.

## Supplementary Materials

The following supporting information can be downloaded at: https://www.mdpi.com/article/10.3390/antiox11101883/s1, Table S1: Primary antibodies used in the present study and Figure S1: Full-length gel images of Western blot data in Figure 3C, Figure 4C, and Figure 5C.
