# Supplementary material for: Sp1-Mediated Prdx6 Upregulation Leads to Clasmatodendrosis by Increasing Its aiPLA2 Activity in the CA1 Astrocytes in Chronic Epilepsy Rats
Source: Antioxidants (Basel). 2022 Sep 23;11(10):1883. doi: 10.3390/antiox11101883 (PMC9598987; doi:10.3390/antiox11101883)
Supplement: Supplementary file 1 [file antioxidants-11-01883-s001.zip › antioxidants-1871815-supplementary.pdf]

Supplementary information

Sp1-mediated Prdx6 upregulation leads to clasmatodendrosis by increasing its aiPLA2 activity in the CA1 astrocytes in chronic epilepsy rats

Ji-Eun Kim[1,2], Duk-Shin Lee[1,2] and Tae-Cheon Kang[1,2]*

[1] Department of Anatomy and Neurobiology, College of Medicine, Hallym University, Chuncheon 24252, South Korea

[2] Institute of Epilepsy Research, College of Medicine, Hallym University, Chuncheon 24252, South Korea

* Correspondence to: T. -C. Kang, Department of Anatomy and Neurobiology, College of Medicine, Hallym University, Chuncheon, Kangwon-Do 24252, South Korea; Tel: +82-33-248-2524; Fax: +82-33-248-2525; E-mail: tckang@hallym.ac.kr

Supplementary Table S1. Primary antibodies used in the present study.

Antigen	Host	Manufacturer (catalog number)	Dilution used
Prdx6	Sheep	Biosensis (S-073-100)	1:1,000 (IH)<br>1:2,000 (WB)
Nrf2	Rabbit	Invitrogen (PA5-88084)	1:100 (IH)<br>1:2,000 (WB)
LAMP1	Rabbit	Abcam (#ab24170)	1:100 (IH)
GFAP	Mouse	Millipore (#MAB3402)	1:2,000 (IH)
Sp1	Rabbit	Elapscience (ENT4372)	1:100 (IH)<br>1:1,000 (WB)
β-actin	Mouse	Sigma (#A5316)	1:5,000 (WB)

IH: Immunohistochemistry; WB: Western blot

Fig. 3C

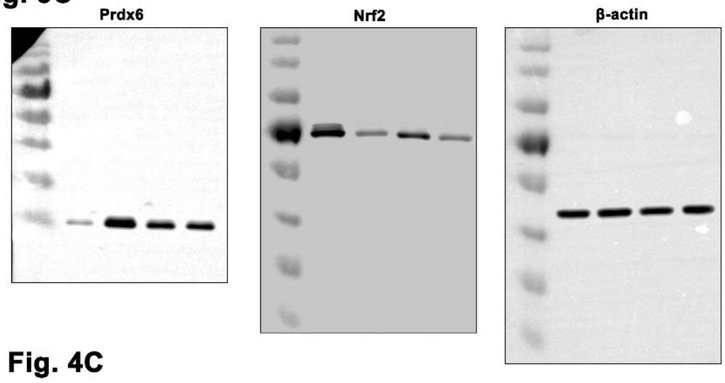

Fig. 4C

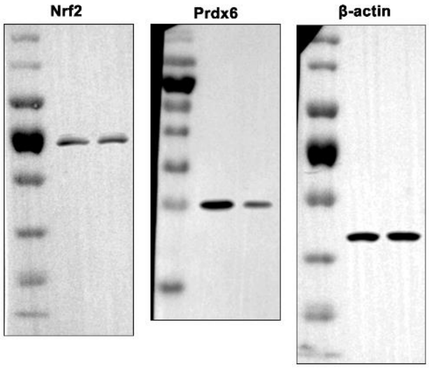

Fig. 5C

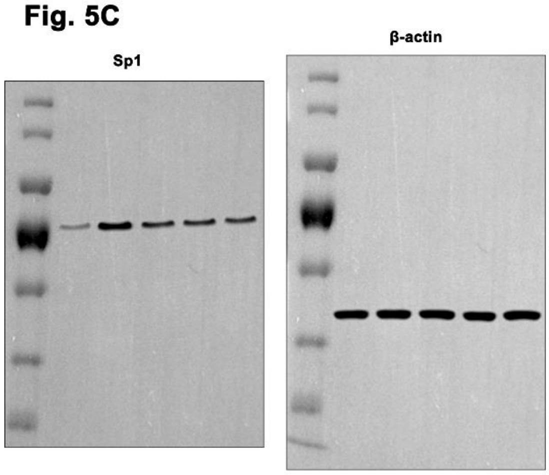

Supplementary Figure S1. Full-length gel images of Western blot data in Figure 3C, 4C and 5C.
